# Generation of a lung squamous cell carcinoma three-dimensional culture model with keratinizing structures

**DOI:** 10.1038/s41598-021-03708-8

**Published:** 2021-12-21

**Authors:** Shigeto Kawai, Kiyotaka Nakano, Keiichi Tamai, Etsuko Fujii, Mimori Yamada, Hiroshi Komoda, Hirofumi Sakumoto, Osamu Natori, Masami Suzuki

**Affiliations:** 1 Department for Research Division 1, Forerunner Pharma Research Co., Ltd., 5–1–1 Tsukiji, Chuo-ku, Tokyo, 104–0045 Japan; 2 Translational Research Division, Chugai Pharmaceutical Co., Ltd., 5–1–1 Tsukiji, Chuo-ku, Tokyo, 104–0045 Japan; 3 Division of Cancer Stem Cell, Miyagi Cancer Center Research Institute, 47–1 Nodayama, Medeshima-Shiode, Natori, Miyagi 981–1293 Japan; 4 Research Division, Chugai Pharmaceutical Co., Ltd., 1–135 Komakado, Gotemba, Shizuoka 412–8513 Japan

**Keywords:** Non-small-cell lung cancer, Tumour heterogeneity

## Abstract

Tumor nests in lung squamous cell carcinoma (LUSC) have a hierarchical structure resembling squamous epithelium. The nests consist of basal-like cells on the periphery and layers of keratinocyte-like cells that differentiate towards the center of the nest, forming keratin pearls. Reproducing this spatial heterogeneity in in vitro models would be useful for understanding the biology of LUSC. Here, we established a three-dimensional (3D) culture model with a squamous epithelial structure using LUSC cell lines PLR327F-LD41 and MCC001F, established in-house. When PLR327F-LD41 cells were cultured in a mixture of Matrigel and collagen I, they generated 3D colonies (designated cancer organoids, or COs) with involucrin (IVL)-positive keratinizing cells in the center (IVL^inner^ COs). COs with uniform size were generated by seeding PLR327F-LD41 cells in a form of small cell aggregates. Since Notch signaling induces the differentiation of squamous epithelium, we confirmed the effect of γ-secretase inhibitor in inhibiting Notch signaling in IVL^inner^ COs. Surprisingly, γ-secretase inhibitor did not block induction of IVL-positive cells; however, cells residing between the CK5-positive basal-like layer and IVL-positive layer decreased significantly. Thus, our 3D culture model with uniform size and structure promises to be a useful tool for elucidating the biology of LUSC and for screening drug-candidates.

## Introduction

Non-small cell lung cancer (NSCLC) is one of the most common causes of cancer death worldwide, major subtypes being lung squamous cell carcinoma (LUSC) and lung adenocarcinoma^1^. In advanced stage lung adenocarcinoma, in which systemic therapy is required, molecular targeted agents inhibiting tyrosine kinases such as EGFR or ALK are effective. In contrast, the major therapeutic options for LUSC are chemotherapy, represented by platinum doublet, or chemoradiotherapy^2^. Immune checkpoint inhibitors are also increasingly used in NSCLC; however, patients suffering from a lack of efficacy are common^1^. Therefore, novel therapeutic options are still needed for NSCLC, especially LUSC. LUSC is classified into keratinizing, nonkeratinizing, and basaloid subtypes^3^. In the keratinizing subtype, a hierarchy of cancer cells from less differentiated basal-like cells to differentiated keratinizing cell constitutes tissue structures referred to as keratin pearls^4^. Since therapy-resistant subpopulations are a major challenge in cancer therapy^5^, understanding the hierarchical differentiation of LUSC would lead to discovery of novel therapeutic options.

The tumour microenvironment contributes to the generation of hierarchical structure in tumour tissues^5^. One of its components is the extracellular matrix (ECM), e.g., the basement membrane or interstitial ECM^6^. The basement membrane is a thin membrane surrounding epithelial tissues that physically supports tissue architecture and transmits polarisation signals to epithelial cells through the adhesion molecule integrin^7^. Interstitial ECM is a component of stroma found between epithelial tissues which is rich in collagen I (col-I)^8^. In tumours, the basement membrane is often breached by tumour cells invading into stroma whereas fibrillar col-I is observed in fibrotic areas. Thus, the complex conditions in tumour ECM potentially influences the hierarchy of cell differentiation.

Three-dimensional (3D) culture models such as spheroid or air–liquid interface culture are used for the analysis of tumor cell plasticity or influence of ECM because they are thought to be physiologically relevant to in vivo conditions^9–11^. Furthermore, 3D organoid culture, which feature primary tumour cells that can maintain phenotypic characteristics of the original tumour, is being developed for various tumour types, including squamous cell carcinoma of the lung or esophagus^12–16^. Although some of these models exhibit keratinization indicating the hierarchical differentiation of tumour cells, few studies have focused on their plasticity in detail.

Here we developed 3D culture model that retain the hierarchical plasticity of LUSC (designated IVL^inner^ cancer organoid or CO) using tumour cell lines PLR327F-LD41 and MCC001F established from LUSC patient-derived xenografts. IVL^inner^ CO with uniform size and structure was efficiently generated by selecting ECM and inoculating cells in the form of small cell aggregates. As a model case for its application, we examined the effect of a γ-secretase inhibitor that blocks Notch signaling during the differentiation process. Due to its uniform size and structure, IVL^inner^ CO is expected to be useful for the biological analysis of LUSC focusing on hierarchical plasticity as well as for screening drug-candidates.

## Results

### PLR327F-LD41 cells formed xenograft tumours showing a hierarchical structure with CK5-positive basal-like cells and keratinizing cells

**Figure 1:**
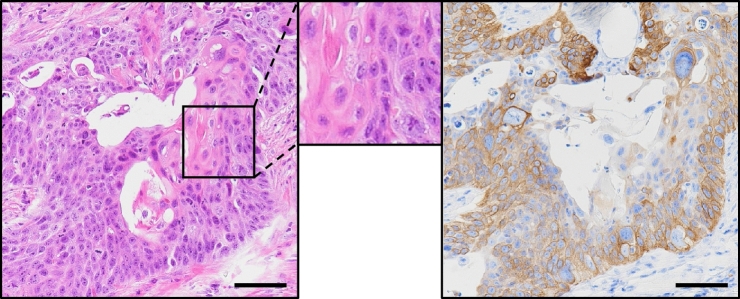
PLR327F-LD41 cells generate tumours with a keratinizing structure in NOG mice. PLR327F-LD41 cells were inoculated subcutaneously into NOG mice. HE stain (left), CK5 immunostaining (right). Inset indicates area of keratinizing structure by histological analysis. Scale bars = 200 μm.

In order to use PLR327F-LD41 cells as the source material for developing 3D culture model, we first evaluated their potential to differentiate and form keratinizing structures. When the cells were inoculated subcutaneously in NOD/Shi-scid, IL-2RγKO Jic (NOG) mice, they formed tumours with a keratinizing structure in the center of the tumour nests evident by hematoxylin and eosin (HE) staining (Fig. 1). Furthermore, basal-like cells located at the periphery expressed basal-cell marker CK5 (Fig. 1). Therefore, PLR327F-LD41 cells were confirmed to possess the potential to generate keratinizing structures characteristic of LUSC and were deemed feasible for generating in vitro culture models with hierarchical differentiation.

### Selection of ECM for 3D culture of PLR327F-LD41 cells

**Figure 2:**
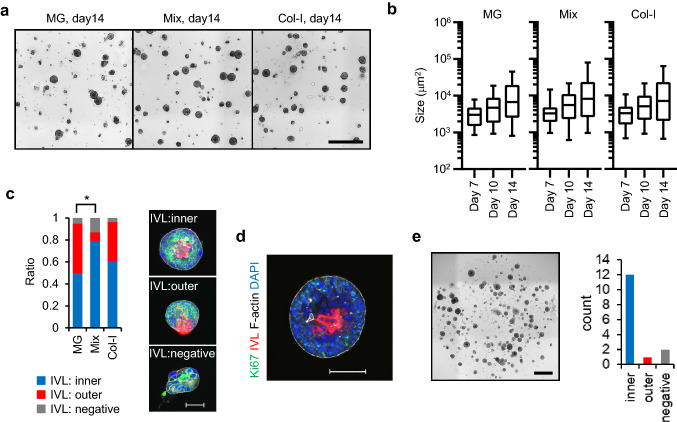
Generation of PLR327F-LD41 single cell-derived 3D culture models. (**a**) Representative bright field images of COs generated by culturing single cells of PLR327F-LD41 in MG, Mix-gel, or col-I for 14 days. Scale bar = 1 mm. (**b**) Growth of COs was determined by analysing the size of each CO from day 7 to day 14. Size (µm^2^) of COs with a major axis longer than 40 µm is shown in box plot. The number of COs analysed was between 90 and 209 for each sample. (**c**) Whole mount immunostaining of Day 14 COs with anti-CK5 antibody (green), anti-IVL antibody (red), phalloidin (white), and DAPI (blue). Scale bar = 100 μm. Each CO was classified by the staining pattern of IVL as IVL^inner^, IVL^outer^, or IVL^negative^. Results from 3 independent experiments were analysed to determine the difference in the ratio of IVL^inner^ COs. Twenty-six to 37 COs were analysed for each sample. *p < 0.05, Tukey–Kramer HSD test. (**d**) Whole mount immunostaining of CO cultured in Mix-gel for 14 days with anti-Ki-67 antibody, anti-IVL antibody, phalloidin, and DAPI. Scale bar = 100 μm. (**e**) COs in Mix-gel were passaged 3 times by mechanically fragmenting the COs and cultured for a total of 45 days. Representative bright field image of the Day 45 COs is shown. Scale bar = 1 mm. Classification of IVL staining pattern was determined for 15 COs. Bright field images and the size analysis of COs were obtained by cellSens Dimension software (version 2.1, Olympus, https://www.olympus-lifescience.com/en/micro/). Whole mount immunostaining images were obtained by NIS Elements HC software (version 5.02, Nikon, https://www.microscope.healthcare.nikon.com/en_EU/).

We attempted to generate a PLR327F-LD41 3D culture model replicating the keratinizing structure found in the xenograft model. For the 3D culture, culture medium with no added serum or growth factors was used to promote differentiation of the cells. ROCK inhibitor Y-27632 was added for the first 3 days to block anoikis of the seeded single cells. For ECM, basement membrane extract Matrigel, col-I, and a 1:1 mixture of them (Mix-gel) was compared. When PLR327F-LD41 cells were cultured in these ECMs for 14 days, 3D colonies or COs developed in all 3 conditions at a similar growth rate (Fig. 2a,b, Supplemental Fig. S1a). Next, we determined the structure of each CO by immunostaining for CK5 and involucrin (IVL) (Fig. 2c, Supplemental Fig. S1b). Based on the results, COs were categorized into 3 types; IVL^inner^ COs, accumulation of IVL-positive cells inside the COs; IVL^outer^ COs, IVL-positive cells residing also at the margin; IVL^negative^ COs, absence of IVL-positive cells. We focused on IVL^inner^ COs since the structure was deemed to reflect the keratinizing structure of the xenograft model. Since IVL^inner^ COs were most frequently observed in Mix-gel, we selected Mix-gel for the subsequent experiments.

Undifferentiated basal cells contribute to cell growth in stratified squamous epithelium. In IVL^inner^ COs, Ki-67-positive cycling cells were localized in the periphery, indicating the outward growth of COs (Fig. 2d). Since growth factors were not added to the culture medium, we determined whether this cell growth could be maintained for the longer period of 45 days by passaging the COs in Mix-gel using mechanical fragmentation (Fig. 2e). During this culture period, the growth of the COs remained constant and IVL^inner^ structure was also maintained at the end of the culture (Fig. 2e). These results indicate that PLR327F-LD41 cells stably form the IVL^inner^ CO structure under this culture condition.

### Optimizing generation of IVL^inner^ COs

**Figure 3:**
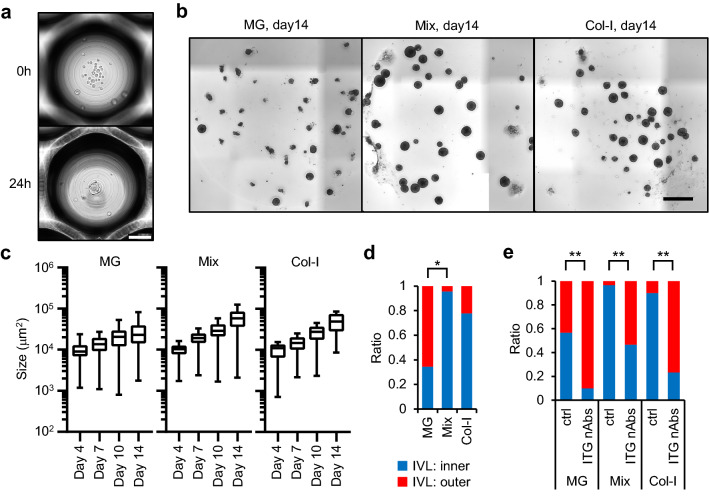
PLR327F-LD41 multicellular spheres generate uniform 3D cultures. (**a**) PLR327F-LD41 cells were cultured in Corning Elplasia microcavity plates for 24 h to form multicellular spheres. Representative pre- and post-culture images are shown. Scale bar = 100 µm. (**b**) Representative bright field images of COs generated by culturing PLR327F-LD41 spheres in MG, Mix-gel, or col-I for 14 days. Scale bar = 1 mm. (**c**) Growth of COs was determined by analysing the size of each CO from day 4 to day 14. Size (µm^2^) of COs with major axis longer than 40 µm are shown in box plot. The number of COs analysed was between 35 and 52 for each sample. (**d**) Results from 3 independent experiments were analysed to determine the difference in the ratio of IVL^inner^ COs. Thirty or 31 COs were analysed for each sample. IVL^negative^ CO was not observed. *p < 0.05, Tukey–Kramer HSD test. (**e**) PLR327F-LD41 spheres were cultured in MG, Mix-gel, or col-I for 14 days. Anti-integrin β1 and β4 neutralising antibodies were added to the culture from Day 7 to Day 14 at 10 µg/mL for each antibody. IVL staining pattern was analysed by whole mount immunostaining. **p < 0.01, Fisher’s exact test. Bright field images and the size analysis of COs were obtained by cellSens Dimension software (version 2.1, Olympus, https://www.olympus-lifescience.com/en/micro/). Whole mount immunostaining images were obtained by NIS Elements HC software (version 5.02, Nikon, https://www.microscope.healthcare.nikon.com/en_EU/).

Although Mix-gel supported the generation of IVL^inner^ COs, their sizes varied to a great extent. To establish a more homogenous culture, PLR327F-LD41 cells were inoculated in the ECM in the form of small multicellular aggregates or spheres. Spheres composed of 30–40 cells were generated by culturing PLR327F-LD41 cells in a microcavity plate (Elplasia, Corning) for 24 h (Fig. 3a). Then they were collected and seeded in the ECM. The ROCK inhibitor was not added. As a result, COs with more uniform size were generated and, notably, more than 95% of COs in Mix-gel were classified as IVL^inner^ COs (Fig. 3b,c,d, Supplemental Fig. S2a,b).

Integrins are responsible for recognizing ECM and determining the polarity of the cell. We determined the dependence of IVL^inner^ COs on integrins by blocking their function by adding integrin β1 and β4 neutralising antibodies to the culture. Integrin neutralization decreased the ratio of IVL^inner^ COs significantly in all 3 types of ECM (Fig. 3e). The size of the COs was decreased in Mix-gel and col-I (Supplemental Fig. S2c,d). Expression of integrin β1 and β4 at the margin of the COs was confirmed by whole mount immunostaining, which suggested their interaction with the ECM (Supplemental Fig. S2e). From these results, integrin-dependent attachment to the ECM was deemed critical for the formation of IVL^inner^ COs, and also for the growth of COs in Mix-gel and col-I.

**Figure 4:**
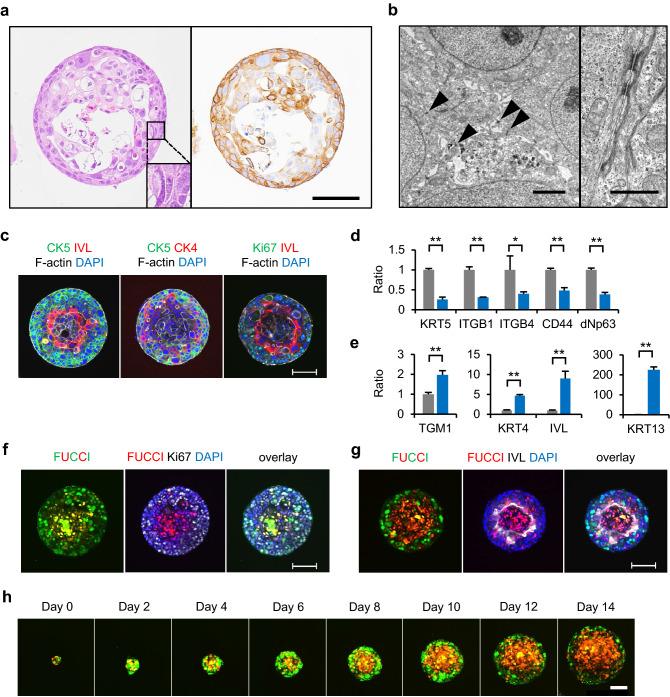
Characterization of the COs generated from PLR327F-LD41 spheres in Mix-gel. PLR327F-LD41 spheres were cultured for 14 days in Mix-gel. COs were characterised by histopathology and marker expression as well as cell growth. (**a**) Sections of COs were stained with HE (left) or anti-CK5 antibody (right). Scale bar = 200 μm. (**b**) Transmission electron microscopy of COs. Arrowheads, desmosomes. Scale bar = 2 μm (left) or 1 μm (right). (**c**) Whole mount immunostaining with anti-CK5 antibody or anti-Ki-67 antibody, anti-IVL antibody or anti-CK4 antibody, and phalloidin and DAPI. (**d**, **e**) Expression of basal cell markers *KRT5*, *ITGB1*, *ITGB4*, *CD44*, and *ΔNp63* (**d**) or differentiation markers *TGM1*, *KRT4*, *KRT13*, and *IVL* by qPCR (**e**). Gray bars, 2D control; blue bars, CO. The relative expression ratio was calculated in relation to a 2D control. Data represent mean + s.d. (n = 3). *p < 0.05; **p < 0.01, Student *t* test. (**f**, **g**) COs generated with PLR327F-LD41-Fucci cells were stained with anti-Ki-67 antibody or anti-IVL antibody and DAPI. Fucci-S/G_2_/M Green and Fucci-G_1_ Orange were overlaid with Ki-67 staining (**f**) or IVL staining (**g**). (**h**) Confocal live-imaging of Fucci-S/G_2_/M Green and Fucci-G_1_ Orange in CO generated with PLR327F-LD41-Fucci cells. Consecutive observation from Day 0 to Day 14. Scale bars = 100 μm (**c**, **f**, **g**, **h**). Whole mount immunostaining images were obtained by NIS Elements HC software (version 5.02, Nikon, https://www.microscope.healthcare.nikon.com/en_EU/). Confocal live-imaging was analysed by CQ1 Software (version 1.06, Yokogawa Electric, https://www.yokogawa.com/solutions/products-platforms/life-science/).

Next, Day 14 IVL^inner^ COs generated from spheres in Mix-gel were characterised in detail. Histopathologically, a squamous epithelium-like structure was observed with basal-like cells on the periphery and keratinized cells in the center (Fig. 4a). Intercellular bridges were observed in some areas, indicating differentiation to keratinocytes. The presence of CK5-positive cells was confirmed by immunohistochemistry. Transmission electron microscopy revealed well-developed desmosomes with associated tonofilaments (Fig. 4b). Markers for differentiation and cell growth were also determined by whole mount immunostaining and gene expression analysis with quantitative real-time PCR (qPCR) (Fig. 4c–e). CK5-positive cells resided at the outermost part of each CO, CK4-positive cells immediately inside, followed by IVL-positive cells (Fig. 4c). However, the innermost part of COs was mostly negative for IVL. Cells positive for Ki-67 localized mainly to the periphery of COs mutually exclusive with the IVL layer (Fig. 4c). Downregulation of basal-cell markers *KRT5*, *ITGB1*, *ITGB4*, *CD44*, and *ΔNp63*, and upregulation of differentiation markers *TGM1*, *KRT4*, *KRT13*, and *IVL* in IVL^inner^ COs were confirmed by qPCR (Fig. 4d,e). These results indicated the hierarchical differentiation of PLR327F-LD41 cells in IVL^inner^ COs.

Since it was speculated that the growth of PLR327F-LD41 cells is arrested and differentiate towards the center of COs, we visualized cell-cycle progression by transfecting PLR327F-LD41 cells with Fucci probes^17^ (designated PLR327F-LD41-Fucci). In IVL^inner^ COs generated with PLR327F-LD41-Fucci cells, G1-phase cells expressing Fucci red fluorescence and negative for Ki-67 accumulated inside each CO (Fig. 4f). Furthermore, the region of accumulation overlapped with the IVL positive layer indicating a synchronic growth arrest and differentiation in the COs (Fig. 4g). Finally, we observed the temporal process of this growth arrest using time-lapse confocal microscopy (Fig. 4h, Supplemental movie). Accumulation of the G1-phase cells within the COs was evident as early as Day 4, and the area increased steadily with the growth of COs. Interestingly, the G1-phase cells seldom changed their location, presumably because of its loss of motility and/or viability.

### Notch signaling regulates the differentiation hierarchy of IVL^inner^ COs

**Figure 5:**
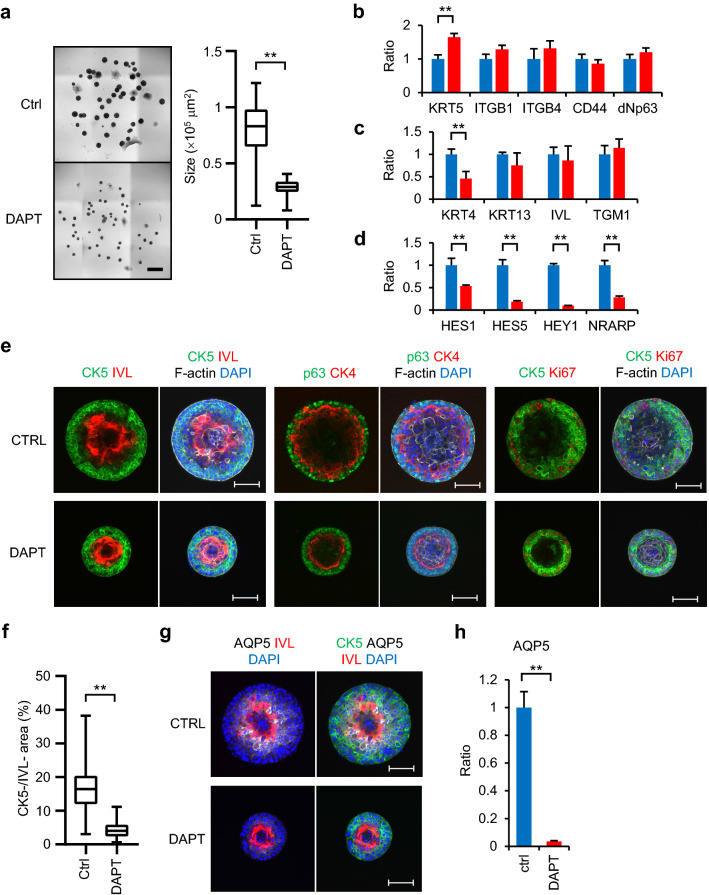
Involvement of Notch signaling in differentiation of PLR327F-LD41 IVL^inner^ CO. PLR327F-LD41 spheres were seeded in Mix-gel and cultured for 14 days with 10 µM DAPT or DMSO control. (**a**) Representative bright field images of COs. Scale bar = 1 mm. Difference in size of COs between DMSO control and DAPT-treated groups. Forty-four or 45 COs were analysed for each sample. **p < 0.01, Student *t* test. (**b**, **c**, **d**) Expression of basal cell markers *KRT5*, *ITGB1*, *ITGB4*, *CD44*, and *ΔNp63* (**b**), differentiation markers *TGM1*, *KRT4*, *KRT13*, and *IVL* (**c**), and Notch signaling markers *HES1*, *HES5*, *HEY1*, and *NRARP* (**d**) by qPCR. Blue bars, DMSO control; red bars, DAPT-treated CO. The relative expression ratio was calculated in relation to DMSO control. Data represent mean + s.d. (n = 3). **p < 0.01, Student *t* test. (**e**) Whole mount immunostaining for CK5 and IVL, p63 and CK4, or CK5 and Ki-67. Phalloidin and DAPI were stained with every sample. Scale bars = 100 μm. (**f**) Area negative for both CK5 and IVL was calculated for DMSO control and DAPT-treated groups. Twenty-seven or 29 COs were analysed for each sample. **p < 0.01, Student *t* test. (**g**) Whole mount immunostaining for CK5 (green), IVL (red), AQP5 (white), and DAPI (blue). Scale bars = 100 μm. (**h**) Expression of *AQP5* by qPCR. The relative expression ratio was calculated in relation to DMSO control. Data represent mean + s.d. (n = 3). **p < 0.01, Student *t* test. Bright field images and the size analysis of COs were obtained by cellSens Dimension software (version 2.1, Olympus, https://www.olympus-lifescience.com/en/micro/). Whole mount immunostaining images and the ratio of CK5^neg^/IVL^neg^ area of COs were obtained by NIS Elements HC software (version 5.02, Nikon, https://www.microscope.healthcare.nikon.com/en_EU/).

Differentiation of normal epidermis is induced by Notch signaling^18^. The Notch signaling pathway is also upregulated in LUSC^19^. Therefore, we aimed to determine if Notch signaling was involved in differentiation of IVL^inner^ COs. PLR327F-LD41 spheres were seeded in Mix-gel and cultured with the γ-secretase inhibitor DAPT for 14 days to block Notch signaling, and marker expression was determined by qPCR (Fig. 5a–d). The growth of COs was significantly inhibited by DAPT (Fig. 5a). Among the basal-cell markers and differentiation markers tested, *KRT5* was upregulated and *KRT4* was downregulated (Fig. 5b,c). Inhibition of Notch signaling was confirmed by the downregulation of Notch signaling markers *HES1*, *HES5*, *HEY1*, and *NRARP* (Fig. 5d). Next, the differentiation hierarchy of the cells in COs was determined by whole mount immunostaining (Fig. 5e). COs treated with DAPT retained the IVL^inner^ phenotype and staining pattern of basal-cell markers CK5 and p63, and differentiation markers IVL and CK4. Growth marker Ki-67 was grossly similar to that of control IVL^inner^ COs. Histopathological analysis indicated increased polarisation of the basal-like cells and keratinization of the differentiated cells in COs treated with DAPT (Supplemental Fig. S3a,b). However, when the spatial relationship of CK5-positive and IVL-positive layers was elucidated in detail, the two layers were closer to each other in DAPT-treated COs with decreased negative areas for both CK5 and IVL (Fig. 5f, Supplemental Fig. S3c,d). We searched for markers which can specifically detect this CK5/IVL double-negative population and focused on aquaporin-5 (AQP5) which is expressed in normal epidermis and epithelial cells of airways, and in squamous cell carcinoma of the oral cavity^20,21^. AQP5 clearly localized to the CK5/IVL double-negative population in IVL^inner^ COs (Fig. 5g). Furthermore, its expression in protein and mRNA was significantly decreased by DAPT treatment (Fig. 5g,h). Therefore, we identified CK5, AQP5, and IVL as markers to determine the hierarchical differentiation of PLR327F-LD41 cells.

### Generation of IVL^inner^ COs with LUSC cell line MCC001F

**Figure 6:**
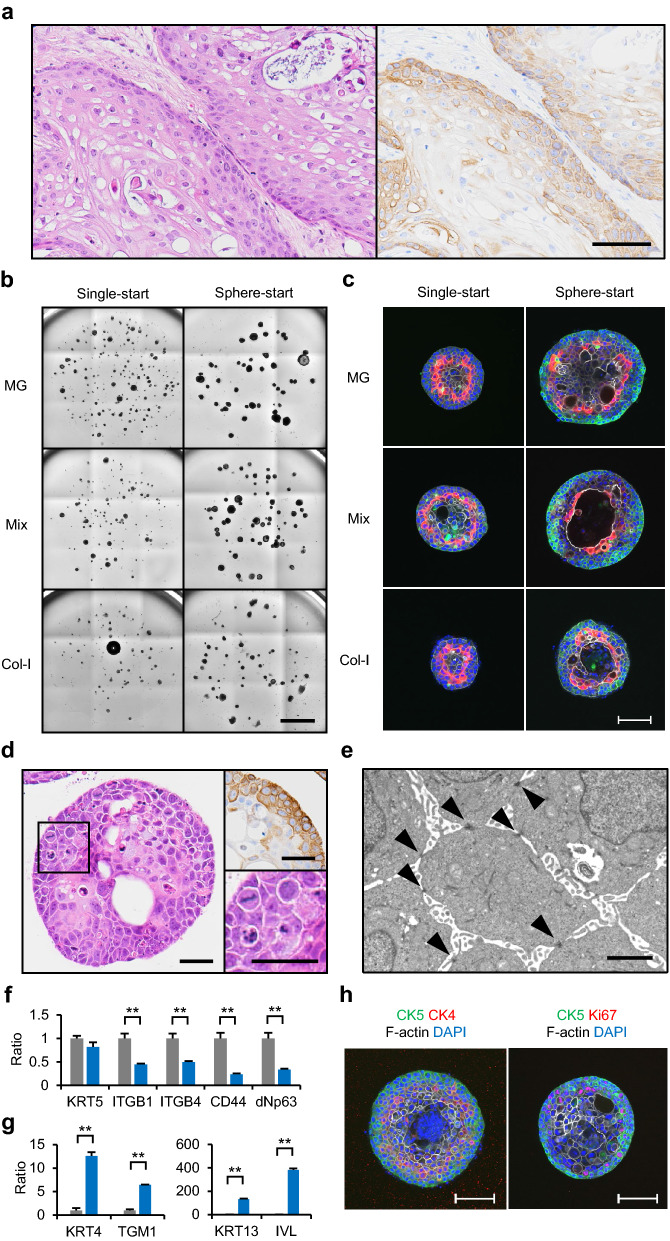
Generation of 3D culture models with MCC001F cells. (**a**) MCC001F cells were inoculated subcutaneously into NOG mice to determine their potential to form tumours with the characteristic structure of LUSC. HE stain (left) and CK5 immunostaining (right) of the xenograft tumours were determined. Scale bar = 200 μm. (**b**) MCC001F single cells or spheres were seeded in MG, Mix-gel, or col-I and cultured for 14 days. Representative bright field images are shown. Scale bar = 2 mm. (**c**) Whole mount immunostaining of MCC001F Day14 COs with anti-CK5 antibody (green), anti-IVL antibody (red), phalloidin (white), and DAPI (blue). Scale bar = 100 μm. (**d**–**h**) MCC001F spheres were seeded in Mix-gel and cultured for 14 days. Sections of COs were stained with HE (left and lower right) or anti-CK5 antibody (upper right). Scale bars = 100 μm (**d**). Transmission electron microscopy of COs. Arrowheads, desmosomes. Scale bar = 2 μm (**e**). Expression of basal cell markers *KRT5*, *ITGB1*, *ITGB4*, *CD44*, and *ΔNp63* (**f**) or differentiation markers *TGM1*, *KRT4*, *KRT13*, and *IVL* by qPCR (**g**). Gray bars, 2D control; blue bars, CO. The relative expression ratio was calculated in relation to a 2D control. Data represent mean + s.d. (n = 3). **p < 0.01, Student *t* test (**f**, **g**). Whole mount immunostaining of COs for CK5 (green), CK4 (red), phalloidin (white), and DAPI (blue) or CK5 (green), Ki-67 (red), phalloidin (white), and DAPI (blue). Scale bar = 100 μm (**h**). Bright field images of COs were obtained by cellSens Dimension software (version 2.1, Olympus, https://www.olympus-lifescience.com/en/micro/). Whole mount immunostaining images were obtained by NIS Elements HC software (version 5.02, Nikon, https://www.microscope.healthcare.nikon.com/en_EU/).

General applicability of the culture method to IVL^inner^ COs was tested with another LUSC cell line, MCC001F. MCC001F was established from LUSC patient-derived xenograft MCC001 similarly to PLR327F. MCC001F cells formed xenograft tumours with keratinized cells in the center of tumour nests and CK5 expression in the basal-like cell layers at the stromal interface (Fig. 6a). When MCC001F cells were cultured in MG, col-I, or Mix-gel, CO developed in all conditions, although its size was significantly small with col-I when MCC001F spheres were used as a starting material (Fig. 6b, Supplemental Fig. S4a,b). In contrast to PLR327F-LD41 cells, IVL^inner^ COs were frequently observed in all 3 ECMs (Fig. 6c, Supplemental Fig. S4c,d). Large lumens were occasionally detected inside the COs regardless of the ECM (Fig. 6c). For further characterization, we cultured MCC001F spheres in Mix-gel for 14 days as with PLR327F-LD41. Keratinization in the center and intercellular bridges as well as the presence of CK5 expression were detected by histopathology and immunohistochemistry as was seen with PLR327F-LD41 cells (Fig. 6d). Desmosomal junctions were also detected by transmission electron microscopy (Fig. 6d,e). Finally, downregulation of basal-cell markers *ITGB1*, *ITGB4*, *CD44*, and *ΔNp63*, and upregulation of differentiation markers *KRT4*, *KRT13*, *TGM1*, and *IVL* in IVL^inner^ COs were detected by qPCR (Fig. 6f,g). CK4 positive cells localized adjacent to the CK5 layer and Ki-67 resided in the CK5 layer as with PLR327F-LD41 (Fig. 6h). Thus, it was demonstrated that our culture protocol of seeding tumour cells in spheres in MG, col-I, or Mix-gel, and culturing in medium with no growth factors is an efficient method to induce hierarchical differentiation of LUSC in vitro.

## Discussion

We generated an IVL^inner^ CO that differentiates and keratinizes towards the center using a 3D culture with 2 cell lines, PLR327F-LD41 and MCC001F, which were established from patient-derived xenograft models of LUSC. The structure formed by the IVL^inner^ COs resembled the hierarchical structures of the xenograft tumours, thus were considered useful as models for in vitro analysis of the hierarchical plasticity of the cell lines. As a model case for biological analysis, a γ-secretase inhibitor was found to reduce the CK5/IVL double-negative layer in PLR327F-LD41 IVL^inner^ COs, indicating the involvement of Notch signaling in the formation of the hierarchical structure.

ECM is known to have a significant effect on cell proliferation and differentiation^7^. The epithelial tissue of the body is lined with a basement membrane, and MG that mimics the basement membrane is widely used in organoid culture of normal and tumour tissues. Additionally, since tumour tissues grow to invade the col-I-rich interstitium, interaction with col-I is also important to reproduce tumour phenotypes. With PLR327F-LD41 cells, IVL^inner^ COs could be produced more efficiently with Mix-gel compared with MG or col-I alone. Decrease in the ratio of IVL^inner^ COs by integrin neutralizing antibodies demonstrated that ECM contact via integrins was important to maintain the proper hierarchy. On the other hand, IVL^inner^ COs were formed at a high frequency irrespective of the ECM type with MCC001F cells. Although we could not identify the factor(s) responsible for this ECM-dependence or ECM-independence, it was considered useful to compare ECMs for each cell line used in order to generate 3D culture models with more homogenous structures.

When the cells were seeded in 3D matrices in a sphere state, COs with uniform size and structure could be prepared. For differentiation-related in vitro assays of cell growth inhibition or gene expression analysis, or for high-content imaging, models with uniform characteristics are preferred for their accuracy. In general, sphere culture is used to induce and/or concentrate cancer stem cells^22,23^. If the PLR327F-LD41 spheres also maintained undifferentiated traits, it was assumed to be a valid starting point for initiating 3D culture. Furthermore, we think the omission of ROCK inhibitors is an advantage of sphere-start culture, since ROCK inhibitors could interfere with the intrinsic properties of cancer cells or inhibit their growth^24^. Based on the above, this method of seeding cells in spheres should be effective for preparing a 3D culture model.

In PLR327F-LD41 IVL^inner^ COs, inhibition of Notch signaling reduced the CK5/IVL double-negative cell layers. In clinical squamous cell carcinoma, there is a distance between the basal-like cell layer expressing p63 and the terminally differentiated IVL-positive layer^25^ and Notch signaling is responsible for the growth arrest and differentiation of squamous cell carcinoma through the mutual inhibition of p63 signaling^26^. On the other hand, IVL-positive cells remained unchanged even if Notch signaling was blocked. Therefore, we speculate that there are mechanisms other than Notch signaling involved in the differentiation of PLR327F-LD41 cells. We expect that the in vitro culture model with hierarchical differentiation can elucidate this differentiation mechanism and ultimately clarify the biology of LUSC.

Here we established an in vitro 3D culture model of IVL^inner^ COs that has the ability of hierarchical differentiation using 2 LUSC cell lines. In this model, as it was necessary to properly select the ECM, we established a method to generate COs with uniform size and structure by seeding the cells in a sphere form. Thus, IVL^inner^ CO is expected to be a useful tool for elucidating the biology of LUSC with regards to hierarchical differentiation, as well as for accurate screening for drug-candidates by cell growth inhibition analysis or high-content imaging.

## Methods

### Cell lines

A human LUSC cell line, PLR327F-LD41, was established from LUSC patient-derived xenograft model PLR327^27^ as follows.

To obtain an in vitro cell line, cell suspension of cancer cells from the xenograft was prepared by mincing the tissues, and was incubated in DMEM containing Liberase DH (Roche), DNase I (Roche), and ROCK inhibitor Y-27632 (FUJIFILM Wako Pure Chemical). The cells were adherent-cultured in collagen I-coated flasks (Corning) with StemPro hESC SFM medium (Thermo Fisher Scientific) supplemented with 10 µM Y-27632 at 37 °C under 5% CO_2_. The cells were treated with Accutase (Nacalai Tesque) to generate a single cell suspension at each passage. Mouse MHC-I positive cells were depleted from the culture by flow cytometry (BD FACSAria III, BD Biosciences) at the fourth passage. The established cell line, PLR327F, was further subcloned by limiting dilution to obtain a stable cell line, designated PLR327F-LD41. PLR327F-LD41 cells, designated PLR327F-LD41-Fucci, were further transfected with expression vectors for Fucci-S/G_2_/M Green IRES-Neo and Fucci-G_1_ Orange IRES-Puro (MBL) using a lentiviral vector system and double transfectants were selected by Geneticin (Thermo Fisher Scientific) and Puromycin (Merck).

A human LUSC cell line, MCC001F, was established as follows. LUSC tissue was obtained from a patient, as approved by the ethical committee at Miyagi Cancer Center. Informed consent was obtained from the patient. All methods were performed in accordance with the relevant guidelines and regulations. A xenograft model was generated in NOG mice as with PLR327F. In vitro cell line MCC001 was generated similarly to PLR327F, except that the culture medium consisted of DMEM/F12 medium (Merck) supplemented with 5% fetal bovine serum (Bovogen), 5 µg/mL insulin (Thermo Fisher Scientific), 10 µg/mL transferrin (Corning), 30 nM sodium selenite (Corning), 10 nM hydrocortisone (Merck), 10 nM 17β-Estradiol (Alfa Aesar), 2 mM L-glutamine (Merck), and 10 mM HEPES (Thermo Fisher Scientific). Mouse cells were depleted from the culture using Feeder Removal MicroBeads and LS Columns (Miltenyi Biotec). MCC001 cells were further adapted to StemPro hESC SFM medium supplemented with 10 µM Y-27632 and were designated MCC001F.

### Animal experiments

All animal experiments were conducted at the Central Institute for Experimental Animals (CIEA). PLR327F-LD41 cells or MCC001F cells were dissociated with Accutase (Nacalai Tesque), suspended in StemPro hESC SFM medium with 50% Matrigel (Cat# 354234, Corning), and 1 × 10^5^ cells/100 µL were inoculated subcutaneously to both flanks of a female NOG mouse (CLEA Japan). Xenograft tumours were collected 35 or 48 days after injection of PLR327F-LD41 or MCC001F cells, respectively. Mice were euthanised by exsanguination under isoflurane anesthesia. All animal experiments were reviewed and approved by the Institutional Animal Care and Use Committee at CIEA and Forerunner Pharma Research Co., Ltd., and carried out in accordance with the relevant guidelines and regulations. The study was carried out in accordance with the ARRIVE guidelines.

### 3D cultures

Cells were suspended in either MG (Cat# 356231, Corning), 2.4 mg/mL col-I (Cellmatrix Type I -A, FUJIFILM Wako Pure Chemical), or Mix-gel (1:1 mixture thereof), and 500 cells/50 µL/well was seeded into standard or clear-bottomed 24-well culture plates (glass-bottomed, AGC or film-bottomed, Eppendorf) by inserting a drop of ECM gel in the center of each wells. After polymerisation at 37 °C, a basal medium which consisted of Advanced DMEM/F-12 supplemented with 10 mM HEPES, Penicillin–Streptomycin, 2 mM Glutamax I, 1× N-2 Supplement, 1× B-27 Supplement (all from Thermo Fisher Scientific), and 1 mM N-Acetyl-L-cysteine (Merck) was applied and the plates were incubated at 37 °C under 5% CO_2_. The basal medium was supplemented with 10 µM Y-27632 for the first 3 days. The medium was replaced every 3–4 days. In experiments for long-term 3D cultures, COs in Mix-gel were collected by solubilising the gel by treatment with 1000 PU/mL Dispase I (FUJIFILM Wako Pure Chemical) and 0.1 mg/mL Brightase-C (Nippi) for 30 min at 37 °C. COs were then fragmented by forcing the solution through a 70 µm cell strainer (Greiner) and inoculated to new Mix-gel with ×3–5 dilution. In experiments for sphere-start cultures, cells were cultured overnight in an Elplasia 96-well Ultra-Low Attachment microcavity plates (Corning) in StemPro hESC SFM medium (Thermo Fisher Scientific) supplemented with 10 µM Y-27632 at a cell concentration theoretically generating 33-cell spheres. Spheres were then suspended in MG, col-I, or Mix-gel at 50–100 spheres/50 µL/well and cultured in basal medium as with single cells, except that Y-27632 was not added. Bright-field, 3×3 tiled, Z-stack images were obtained using an inverted microscope with 4× lens (IX83, Olympus). In focus information from each Z-stack image was extracted and combined into a single image by Extended Focal Imaging (EFI), and the area (µm^2^) of each CO was analysed with cellSens Dimension software (version 2.1, Olympus, https://www.olympus-lifescience.com/en/micro/). Objects with major axis of less than 40 µm were omitted from the analysis because of the difficulty in excluding contaminants.

For blocking integrin, 10 µg/mL each of integrin β1 (clone 6S6, Azide Free, Merck) and integrin β4 (clone UM-A9, Azide free, Novus Biologicals) neutralising antibody was added to the culture from Day 7 to Day 14. For Notch signaling inhibition, 10 μM DAPT (Selleck Chemicals) or DMSO control was added to the culture from Day 0 to Day 14.

### Histological examination

Xenograft tumours of NOG mice engrafted with PLR327F-LD41 and MCC001F cells were fixed with 4% paraformaldehyde (PFA) on ice for 24 h and embedded in paraffin using the AMeX method^28^. Thin sections were prepared for histopathology and immunohistochemistry. Histopathologically, HE-stained slides were prepared and read under a light microscope. For immunohistochemical staining of CK5, an anti-human CK5 antibody (clone EP1601Y, Abcam) was applied as the primary antibody after antigen retrieval in Target Retrieval Solution (Dako), followed by a labeled polymer reagent (Envision + HRP anti-rabbit, Dako) as the secondary antibody, and visualised by DAB solution. The slides were counterstained with hematoxylin and read under a light microscope (Eclipse Ni-U microscope equipped with DS-Ri2 digital camera, Nikon).

For the evaluation of 3D cultures, paraffin blocks of Mix-gel-cultured COs were prepared using a method described previously^29^. Briefly, the culture was treated with 0.1 mg/mL Brightase-C for 30 min at 37 °C, mechanically disrupted, fixed with 4% PFA for 2 h at 4 °C, and the gel was further dissociated by pipetting. Then the COs were embedded in paraffin by the AMeX method. HE slides and immunohistochemical staining for CK5 was carried out with the same procedure as the xenograft tumours.

### Whole mount immunostaining for 3D cultures

After removing the medium from the 3D cultures, 400 μL of PBS containing 4% PFA was added and incubated for 2 h at room temperature. The samples were carefully handled thereafter to maintain ECM gel domes. They were then permeabilized by PBS containing 0.5% Triton X-100 overnight at 4 °C and further incubated with BlockAid Blocking Solution (Thermo Fisher Scientific) containing 0.5% Triton X-100 (blocking buffer). Primary antibodies [Alexa Fluor 488-labeled anti-CK5 antibody (clone EP1601Y, Abcam), Janelia Fluor 549- or Alexa Fluor 647-labeled anti-involucrin antibodies (clone SPM259, Novus Biologicals), Alexa Fluor 488-, Alexa Fluor 555-, or Alexa Fluor 647-labeled anti-Ki-67 antibodies (clone B56, BD Biosciences), Alexa Fluor 488-labeled anti-p63 antibody (clone EPR5701, Abcam), Alexa Fluor 647-labeled anti-aquaporin 5 antibody (clone EPR3747, Abcam), Alexa Fluor 488-labeled anti-integrin β1 antibody (clone TS2/16, Biolegend), anti-integrin β4 antibody (clone EPR8559, Abcam), or anti-CK4 antibody (clone 6B10, Abcam)] in blocking buffer were added and incubated for 3–4 days at 4 °C, then washed with PBS containing 0.5% Triton X-100 and 1% BSA and incubated overnight at 4 °C with secondary antibody [Alexa Fluor 555-labeled anti-mouse IgG1 antibody (Thermo Fisher Scientific) or Alexa Fluor Plus 488-labeled anti-rabbit IgG antibody (Thermo Fisher Scientific)] in blocking buffer. The wells were then incubated with SeeDB2G Solutions to clear the 3D cultures^30^. Phalloidin-DyLight 650 and 1 μg/mL DAPI (all Thermo Fisher Scientific) were added to Solution 2 and incubated overnight at 4 °C. Finally, the wells were observed with a confocal fluorescence microscope (A1, Nikon). When staining integrin β1 or β4, COs in Mix-gel or col-I were collected with 0.1 mg/mL Brightase-C and COs in Matrigel were collected after fixing the gel domes with 4% PFA. Saponin, 2%, was used instead of Triton X-100.

Each CO was classified by the staining pattern of IVL in one confocal plane as follows; IVL-accumulation, few or no IVL-positive cells (up to 3 cells) at the periphery of the COs and the majority of IVL-positive cells are located inside the COs as a core or ring-like accumulation; IVL-scattered, IVL-positive cells are scattered across the section (IVL-accumulation and IVL-scattered were grouped as IVL^inner^); IVL^negative^, IVL-positive cells are not observed; IVL^outer^, IVL-positive cells are mainly observed at the periphery. COs with a diameter over 100 µm were analysed. CK5/IVL double negative area (CK5^neg^/IVL^neg^ area, µm^2^) was calculated by subtracting the area positively stained with anti-CK5 and anti-IVL antibodies from the whole CO area as determined by DAPI staining using NIS Elements HC software (version 5.02, Nikon, https://www.microscope.healthcare.nikon.com/en_EU/). The ratio of CK5^neg^/IVL^neg^ to the whole CO area was calculated.

### Confocal live imaging for 3D cultures

Spheres generated with PLR327F-LD41-Fucci cells were suspended in Mix-gel at 100 spheres/50 µL/well, set under a Confocal Quantitative Image Cytometer CQ1 (CQ1 Software version 1.06, Yokogawa Electric, https://www.yokogawa.com/solutions/products-platforms/life-science/), and bright-field and confocal fluorescent images (excitation 488 and 561 nm) were monitored at 8-h intervals from Day 0 to Day 14.

### Transmission electron microscopy for 3D cultures

3D cultures in Mix-gel were fixed with half Karnovsky solution for 2 h at 4 °C. The gels were cut into pieces of approximately 1 mm^3^, washed with 0.1 M cacodylate buffer, and post-fixed with 1% osmium tetroxide in 0.1 M cacodylate buffer for 1.5 h at 4 °C. The samples were then dehydrated in a series of ethanol solutions, replaced with propylene oxide, and embedded in epoxy resin (Quetol812, Nisshin EM). Resin was heat polymerised. Ultrathin (approximately 70 nm) sections were prepared using a ultramicrotome (Leica EM UC7, Leica Microsystems), double-stained with uranyl acetate and lead citrate, and observed with a transmission electron microscope (HT7700, Hitachi High-Technologies).

### Real-time quantitative PCR

Total RNA was extracted from 3D cultures or from 2D monolayer cells using Trizol (Thermo Fisher Scientific). Complementary DNA was synthesized with a SuperScript III First-Strand Synthesis System (Thermo Fisher Scientific) according to the manufacturer’s instructions. qPCR was performed in duplicate for each gene on a StepOnePlus Real-Time PCR System (Thermo Fisher Scientific) using Power SYBR Green (Thermo Fisher Scientific). *RPS18* was used as an internal control. Fold difference in gene expression was determined by the 2-ΔΔCt method. The sequence of the primers for qPCR are shown in Supplementary Table S1 online.

### Statistical analysis

Statistical analyses were performed using the JMP software (version 11.2.1 or 15.0.0, SAS Institute). The Tukey–Kramer HSD test was employed to determine the statistical significance of the differences in CO size between MG, col-I, and Mix-gels at Day 14. To determine the statistical significance of the ratio of IVL^inner^ COs between MG, col-I, and Mix-gels, experiments comparing each ECM were done 3 times and the results were determined by the Tukey–Kramer HSD test. To determine the effects of integrin neutralising antibodies on the ratio of IVL^inner^ COs, 30 COs each were analysed for test and control groups, and statistical significance was determined by the Fisher’s exact test. Difference in the size of the CO or CK5^neg^/IVL^neg^ area (%) after treatment with integrin neutralising antibodies or DAPT between test and control groups was determined by the Student *t* test, which was also employed to determine statistical significance in gene expression analysis. A *p* value of < 0.05 was considered statistically significant.

## Supplementary Information

Supplementary Video 1.

Supplementary Information 1.

## Data Availability

All data generated or analysed during this study have been included in this published article.
