# Supplementary material for: Generation of a lung squamous cell carcinoma three-dimensional culture model with keratinizing structures
Source: Sci Rep. 2021 Dec 21;11:24305. doi: 10.1038/s41598-021-03708-8 (PMC8692465; doi:10.1038/s41598-021-03708-8)
Supplement: Supplementary file 2 — Supplementary Information 1. [file 41598_2021_3708_MOESM2_ESM.pdf]

Generation of a lung squamous cell carcinoma three-dimensional culture model with keratinizing structures

Shigeto Kawai[1,2*], Kiyotaka Nakano[1,2], Keiichi Tamai[3], Etsuko Fujii[1,2], Mimori Yamada[1,2], Hiroshi Komoda[1], Hirofumi Sakumoto[1,4], Osamu Natori[1,4] & Masami Suzuki[1,4]

Affiliation:

[1]Department for Research Division 1, Forerunner Pharma Research Co., Ltd., 5–1–1 Tsukiji, Chuo-ku, Tokyo 104–0045, Japan

[2]Translational Research Division, Chugai Pharmaceutical Co., Ltd., 5–1–1 Tsukiji, Chuo-ku, Tokyo 104–0045, Japan

[3]Division of Cancer Stem Cell, Miyagi Cancer Center Research Institute, 47–1 Nodayama, Medeshima-Shiode, Natori, Miyagi 981–1293, Japan

[4]Research Division, Chugai Pharmaceutical Co., Ltd., 1–135 Komakado, Gotemba, Shizuoka 412–8513, Japan

[*]kawaisgt@chugai-pharm.co.jp

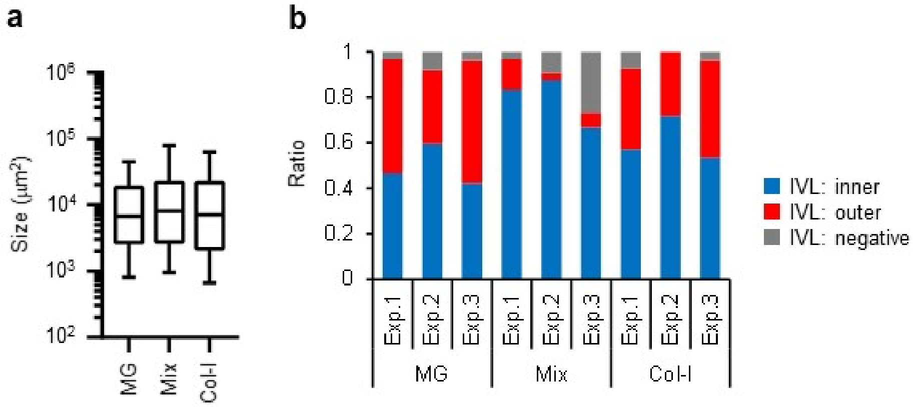

Supplemental Figure S1. Comparison of COs generated from PLR327F-LD41 single cells in MG, Mix-gel, or col-I. Single cells of PLR327F-LD41 were cultured in MG, Mix-gel, or col-I for 14 days. Difference in size of COs was not statistically significant (a). Data of the 3 experiments for classifying IVL staining pattern are indicated (b).

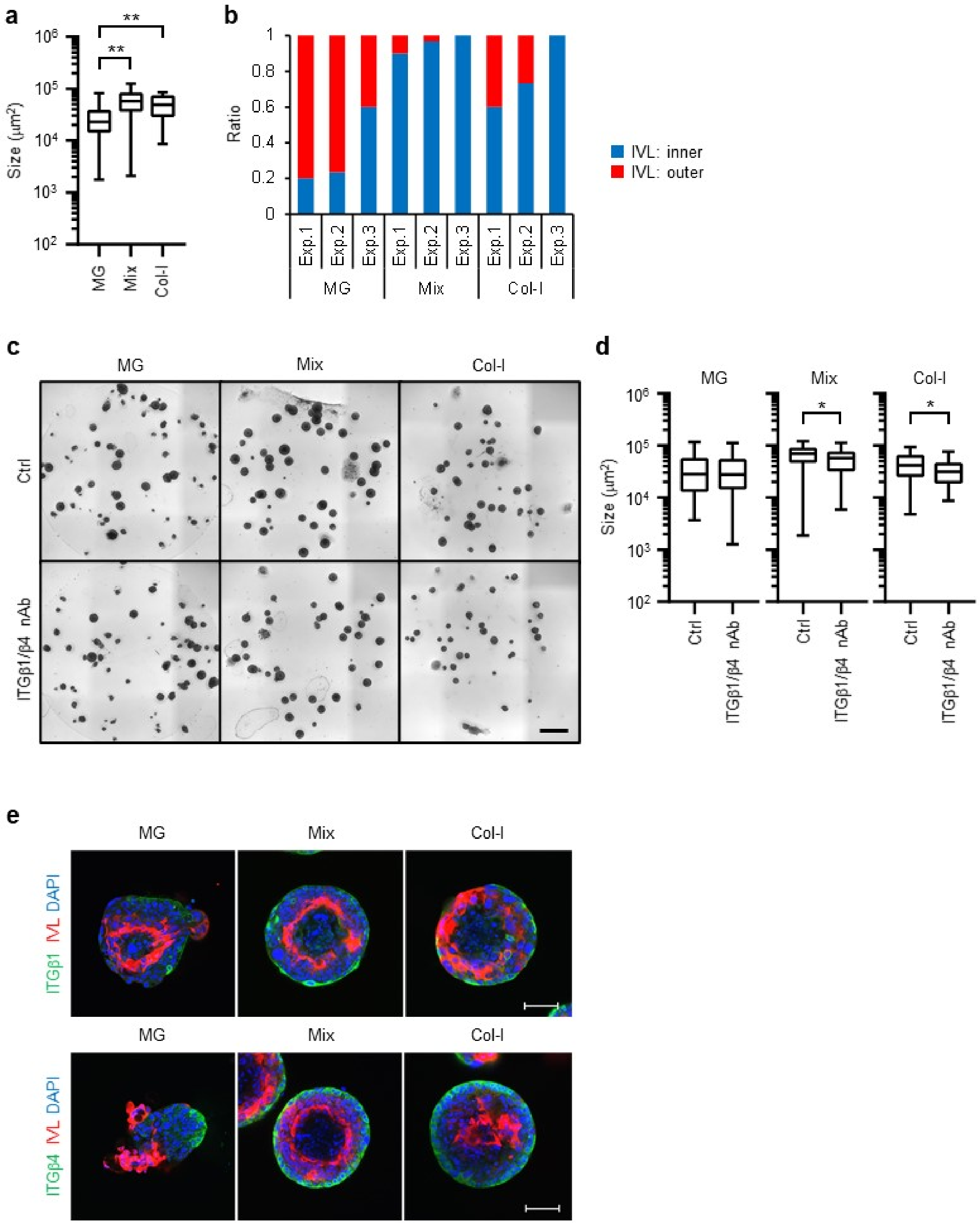

Supplemental Figure S2. Comparison of COs generated from PLR327F-LD41 spheres in MG, Mix-gel, or col-I. PLR327F-LD41 spheres were cultured in MG, Mix-gel, or col-

I for 14 days. (a) Difference in size of COs was analysed by the Tukey-Kramer HSD test. **, $p<0.01$. (b) Data of the 3 experiments for classifying IVL staining patterns is shown. (c) Representative bright field images of Day 14 cultures with or without integrin β1 and β4 neutralising antibodies. Scale bar = 1 mm. (d) Difference in size of COs between control and integrin β1 and β4 neutralising antibody-treated groups. Thirty-eight to 64 COs were analysed for each sample. *, $p<0.05$, Student *t* test. (e) Whole mount immunostaining of Day 14 COs with anti-integrin β1 antibody (upper) or anti-integrin β4 antibody (lower), and anti-IVL antibody and DAPI. Scale bar = 100 μm.

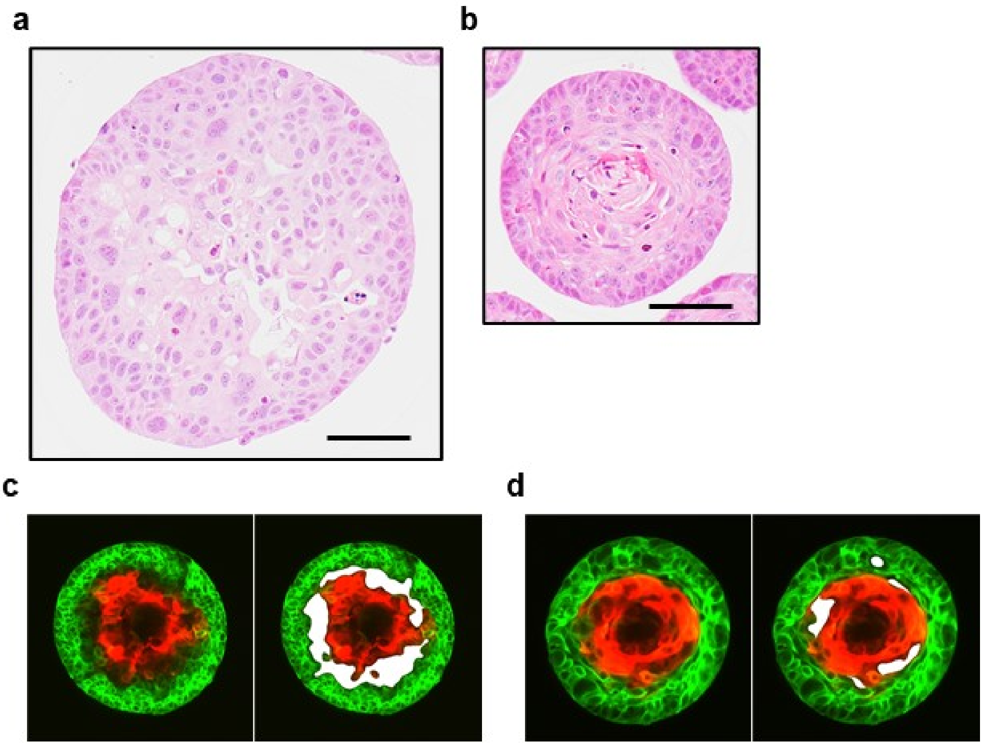

Supplemental Figure S3. Involvement of Notch signaling in differentiation of PLR327F-LD41 IVL^{inner} CO. PLR327F-LD41 spheres were seeded in Mix-gel and cultured for 14 days with 10 μM DAPT or DMSO control. (a,b) Section of the COs cultured with DMSO (a) or DAPT (b) was stained with HE. Scale bar = 200 μm. (c,d) Examples of image analysis of CK5^{negative}/IVL^{negative} area for COs treated with DMSO (c) or DAPT (d). Green, immunostaining with anti-CK5 antibody; red, immunostaining with anti-IVL antibody; white, calculated CK5^{negative}/IVL^{negative} area.

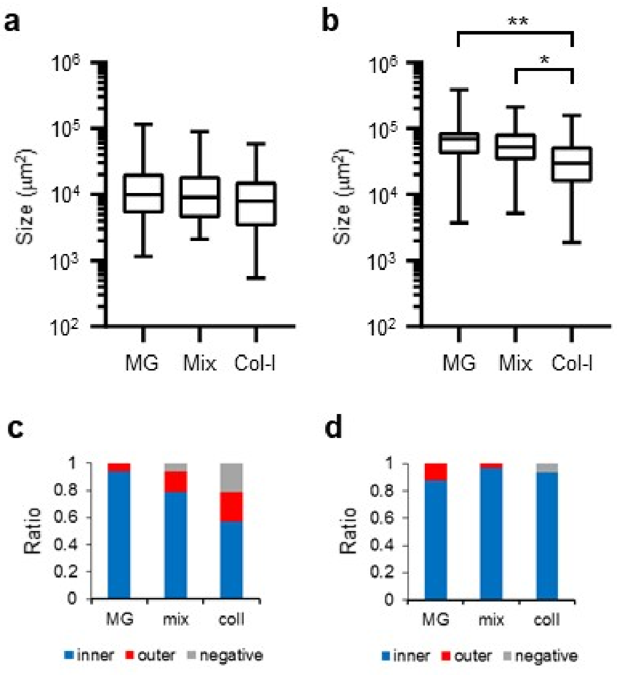

Supplemental Figure S4. Comparison of COs generated from MCC001F single cells and spheres. MCC001F single cells or spheres were seeded in MG, Mix-gel, or col-I and cultured for 14 days. (a,b) Difference in size of COs generated from single cells (a) or spheres (b) was analysed by the Tukey-Kramer HSD test. *, $p<0.05$; **, $p<0.01$. Ninety-one to 173 COs for single cells, 46 to 62 COs for spheres were analysed. (c,d) Classification of IVL staining pattern of COs generated from single cells (c) or spheres (d) analysed by whole mount immunostaining. Twenty-eight to 33 COs were analysed for each sample. Difference between Mix-gel and MG or Mix-gel and col-I was not statistically significant by Fisher's exact test.

Supplemental Table S1. Primers used for quantitative RT-PCR analysis.

Human gene	Forward primer	Reverse primer
KRT5	GAGATCGCCACTTACCGCA	TCCAGAGGAAACACTGCTTGT
ITGB1	AAGCGAAGGCATCCCTGAAA	GTCTACCAACACGCCCTTCA
ITGB4	GGGAAAAAGCAAGACCACACC	CCCTCTGTTCCACCTGCTTC
CD44	GGACAAGTTTTGGTGGCACG	TCCGTCCGAGAGATGCTGTA
dNp63	GAAAACAATGCCCAGACTCAA	TGCGCGTGGTCTGTGTTA
IVL	GCCTCTGCCTCAGCCTTACT	ACTGGAGGAGGAACAGTCTTG
KRT4	GACAGCGTGGAGGACTTCAA	CCTTGTTCAGGTAGGCAGCA
KRT13	GGGACTACAGCCCCTACTACA	TTTTCAATGGTGGCGGTCAG
TGM1	TGCCACACCCCAAGAGACTA	AAGGCGTGTCGTACTTCATGT
HES1	GAAGCACCTCCGGAACCT	GTCACCTCGTTCATGCACTC
HES5	CTACCTGAAGCACAGCAAAG	AGCTTCATCTGCGTGTCG
HEY1	TGGTACCCAGTGCTTTTGAG	CTCCGATAGTCCATAGCAAGG
NRARP	GCGTTGTGAAGGCAACAGAG	GGGAGGCTAAAAAGGGGCAA
AQP5	TCCGGGCTTTCTTCTACGTG	CCCTGCGTTGTGTTGTTGTT
RPS18	GTGCTGCAGCCATGTCTCT	GCAGTGATGGCAAAGGCTAT

Supplemental movie. Live-imaging of CO generated with PLR327F-LD41-Fucci cells. Confocal live-imaging of Fucci-S/G_2/M Green and Fucci-G_1 Orange in CO generated with PLR327F-LD41-Fucci cell spheres in Mix-gel. Images were observed from Day 0 to Day 14 at 8-hour intervals. Scale bar = 100 μm.
